# Acute suppurative thyroiditis with thyroid abscess in adults: clinical presentation, treatment and outcomes

**DOI:** 10.1186/s12902-019-0458-0

**Published:** 2019-12-03

**Authors:** Henrik Falhammar, Göran Wallin, Jan Calissendorff

**Affiliations:** 1 Department of Molecular Medicine and Surgery, Karolinska Institutet, Stockholm, Sweden; 2 Department of Endocrinology Metabolism and Diabetes, Karolinska University Hospital, Stockholm, Sweden; 3 Deptartment of Surgery, Faculty of Medicine and Health, Örebro University, Örebro, Sweden

**Keywords:** Abscess, Infection, Surgery, Antibiotic treatment, Drainage

## Abstract

**Background:**

Abscess in the thyroid gland is a rare but severe infectious disease. The condition can have anatomic or iatrogenic underlying causes. If untreated it could be fatal. Pathogens vary considerably. Treatment is intravenous antibiotics, drainage, and sometimes surgery.

**Methods:**

The electronic medical records of all adult patients with acute thyroiditis 2003–2017 treated at the Karolinska University Hospital (catchment area 2 million) in Sweden were systematically reviewed.

**Results:**

Five patients were found in the catchment area. One patient from another region but known to us was also included. Thus, six patients (aged 28–73 years) were included in the study. Median length of hospital stay was 7.5 days (4–79 days). All were treated with antibiotics (intravenous *n* = 5, oral *n* = 1). Total antibiotic treatment duration was 13.5 days (10–41 days). Blood cultures were positive in three (*streptococcus pneumonia*, *streptococci sanguineous, pepto streptococci*), deep tissue culture in three (*Escherichia coli*, *Candida*, *Hemophilic influenza*) and no positive culture at all in two. Drainage was used in three patients. All patients recovered without recurrences. Surgery was performed twice in the acute phase in one. There was no recurrence during 7 years (3–12) of follow-up, but one patient died after three years (severe heart failure and pneumonia).

**Conclusion:**

Thyroid abscess in adults is extremely rare nowadays in the developed world. With prompt antibiotic therapy, drainage and in some cases thyroidectomy the prognosis seems favourable.

## Introduction

Acute suppurative thyroiditis (AST) is an infection in the thyroid gland progressing to an abscess. This is a serious, rare and potentially fatal condition [1]. Due to the encasement of the gland, its rich blood supply, high iodine content and good lymphatic drainage the thyroid is relatively resistant to most infections [2]. AST comprises < 1% of thyroid diseases [1, 3]. In children, this is often secondary to local congenital anatomic defects as third or fourth branchial arch anomalies [4, 5], or a piriform sinus fistula [6]. Only 8% of AST occurs in adults [7]. Hashimoto’s disease [8], large goiters [9] or thyroid cancer could predispose individuals [10], but AST could also arise by hematogenous or lymphatic spread or by iatrogenic infections after fine needle aspiration biopsy (FNA) [11]. Multiple pathogens have been implicated, mainly *Staphylococcus aureus* [12] and streptococci species [12, 13], but also tuberculosis (sometimes in combination with HIV) [14, 15], brucellosis [16], candida [17], salmonella [18], and anaerobes as *Escherichia coli* [19] and klebsiella [20]. Patients with SLE [21], Hodgkin lymphoma [22] and other immunocompromised conditions [23] may be more vulnerable. Clinically patients with AST have fever, neck pain, sore throat which can be painful during swallowing, dysphagia, increased C-reactive protein (CRP), measurable thyroglobulin and positive cultures from drainage or blood. However, neither elevated CRP nor increased thyroglobulin are diagnostic, but the latter is helpful in identifying the thyroid as a site of inflammation or infection but is not a specific marker for AST [1]. Differential diagnoses are subacute or chronic thyroiditis, neck trauma, thyroid cyst rupture, aggressive thyroid cancer or thyroid lymphoma [1, 24]. A FNA can be used to rule out subacute thyroiditis or malignancy. Treatment is drainage and/or thyroid surgery together with antibiotic therapy, as inadequate treatment of abscesses can result in mortality rates of 12% or more [25, 26].

The aim of this investigation was to explore the incidence of thyroid abscess in a region with well-developed health care in adult patients without anatomical defects or who were immunosuppressed, and to describe the clinical presentation, treatment and outcomes.

## Methods

This was a retrospective investigation of all adult cases with a thyroid abscess between 1 January 2003 and 31 December 2017 conducted at the Karolinska University Hospital in Stockholm, Sweden. The Karolinska University Hospital has a catchment area for highly specialized care and thyroid surgery of more than 2 million inhabitants. We knew of three cases with thyroid abscess since previously. Moreover, all patients with an International Classification of Diseases version 10 (ICD-10) code of E06.0 (acute thyroiditis), E07.1 (dyshormogenic goiter), E07.8 (Other specific disorders of thyroid), and E07.9 (Disorder of thyroid, unspecific) were reviewed, to explore if additional cases could be found, and subsequently two more cases were found. All the relevant electronic medical files of the patients were reviewed manually and all clinical and biochemical data of patients with AST were recorded in detail. The National Population Register was also consulted to find out if the included patients were still alive and the date of death was retrieved if applicable [27]. All hospital admissions and specialist out-patient visits in Sweden are coded with ICD-10 codes by the attending doctor and are thereafter stored in both local and national databases [28]. One of the authors (GW) had reviewed all surgical thyroid patients at the Örebro University Hospital (catchment area 280,000 inhabitants) from 2008 until today, and only one patient with AST from that region was found and was subsequently included. Patients with necrosis and infection post-surgery for thyroid malignancies were excluded, children (patients less than 18 years of age) as well as patients with AST secondary to immunosuppression. Results are presented as median and range.

The study was approved by the Regional Ethical Review Board in Stockholm, Sweden. All patients or their next of kin were contacted and written informed consent was obtained.

## Results

In total six patients with thyroid abscess was included (five females). The abscesses were equally commonly located in each thyroid lobe. Median age at presentation was 51 years (28–73 years). None had third or fourth left branchial cleft anomalies nor was immunosuppressed. All patients were successfully treated with antibiotics for 13.5 days (10–41 days), drainage in three, and surgery was performed twice in the acute phase in one and at a later state in one. The length of hospital stay was 7.5 days (4–79 days). Median follow-up time was 7 years (3–12 years). A detailed description is given in the case presentations below and the clinical data are summarized in Table 1.

**Table 1:** Laboratory measures, clinical signs and cultures in patients with thyroid abscess

	CRP mg/L	ESR mm/hr	Fever ºC	TSH mIU/L	Free T 4 pmol/L	TRAb mIU/L	TPOab kIU/L	Tissue cultures	Blood cultures	FNA
Case 1	180	28–75	40	0.1	15	Neg	Neg	NA	Strep pneumonia	Lymphocytes, granulocytes macrophages
Case 2	328	127	37.9	0.08	57	Neg	NA	E coli	Neg × 6	Granulocytes cell debris
Case 3	104	40	38	1.4	13	< 0.3	45	Neg	Neg x 2	Macrophages giant cells^a^
Case 4	87	26	39	0.9	9	0.9^bb^	0.5^ccc^	Neg	Neg x 2	Granulocytes macrophages
Case 5	426	NA	38.4	NA	NA	NA	NA		Strepto- cocci s	
Case 6	202	NA	39.3	0.1	21	< 0.3		Hemophilus Candida	Peptostrepto- cocci	
Normal	< 3	< 20		0.4–3.5/4.7	8–22	< 0.3	< 0.34			

^a^Three weeks after initial lab tests. ^bb^ref. < 1.0. E/L. ^ccc^ref. < 5.0 kE/L. See text for detail as biochemical references were not identical in all tests

FNA, fine needle aspiration. Neg, negative. NA, not available. s, sanguinis

### Case 1

A 48-year-old woman with hypertension and goitre presented with three weeks of on and off fever together with symptoms from the urinary tract and gut. She presented to the Emergency Department with a fever of 40 °C, cough, tachycardia and pain located to the left ear and to the anterior neck. The goitre was tender and to some extent hard but not fluctuating at palpation. From the left lung crepitations were heard on auscultation. The blood biochemical investigations revealed: ESR 28 mm/hr (normal < 20), CRP 180 mg/L (< 3), alanine aminotransferase 2.25 μkat/L (< 0.76), aspartate aminotransferase 1.31 μkat/L (< 0.61), potassium 3.3 mmol/L (3.6–4.6), TSH 0.1 mIU/L (0.4–4.7), free T4 15 pmol/L (12–23) and free T3 3.9 pmol/L (3.0–6.5). No TRAb or TPOab were detected, and other blood tests were normal as were a chest X-ray. She received treatment with prednisolone 30 mg due to suspicion of a subacute thyroiditis, was discharged due to social reasons, and were planned to return the following day. As fever persisted the next day she was admitted, blood cultures were drawn and a FNA from the thyroid performed. Prednisolone was continued with addition of iv cloxacillin 2 g tds and iv benzylpenicillin 3 g tds. Cytology showed granulocytes and a few lymphocytes together with macrophages, colloid and blood. Blood cultures were positive for streptococcus pneumonia, and cloxacillin and prednisolone were withheld. After six days with improvements, CRP had decreased to 80 mg/L, iv benzylpenicillin was changed to per oral phenoxymethylpenicillin 2 g tds for an additional week. The dental status was normal. The conclusion was sepsis with acute thyroiditis and she was discharged after 12 days. Three weeks later she was well but had a foul taste in mouth. MRI was performed but this could not reveal any fistula or branchial arch anomalies. There had been no recurrence at last follow-up 11 years later.

### Case 2

A 54-year-old male with a previous nephrectomy due to a renal cell carcinoma, and benign prostate hyperplasia was seen regularly by the General Practitioner. He had also had multiple prostate biopsies to exclude malignancy. After such a biopsy, he was treated for a urinary septicaemia. A month after that he got flu like symptoms, exhaustion and headache. Seven weeks after the biopsy he presented to the Emergency Department with fever, sweating, loss of appetite, pain in the throat and a foul metal taste. Examination revealed an enlargement of the right thyroid lobe, palpable lymph glands along the sternocleidomastoid muscle, a tachycardia of 119 beats per minutes and a CRP of 328 mg/L (< 3). He was admitted with a suspected infection or malignancy. CT showed a suspect thyroid abscess in the level of the jugulum with suspicion of mediastinitis. He was treated initially with oral ceftibuten 400 mg once daily which was changed to iv imipenem/cilastatin 1 g tds and iv clindamycin 600 mg qid for five days. An echocardiogram to exclude endocarditis was performed which was normal. At an ultrasound guided puncture of the thyroid, brownish pus was seen, and a thyroid drain was inserted. TSH was 0.08 mIU/L (0.4–3.5) and free T4 57 pmol/L (8–14), and po propranolol 20 mg tds was initiated. TRAb and TPOab were negative. Clinical improvement was noted with a decrease in CRP but also an accentuation of hyperthyroidism after five days, which then gradually subsided. Antibiotics were switched to oral ceftibuten 400 mg od for another two weeks. Blood cultures were negative, as were cultures of mycobacterium, urine, throat and nasopharynx, but deep culture from the thyroid showed two variants of *Escherichia coli*. He lost 8 kg and was hospitalized for seven days but at follow-up had regained weight and thyroid parameters were normalized. He was later lost to follow-up but had not been referred again during the last 10 years and was still alive.

### Case 3

A 73-year-old woman presented to the Emergency Department because of a swollen and tender resistance in the neck for five days, and fever of 38 °C the last two days. She had difficulties to swallow. Two years earlier she had had the same experience, FNA at that time was normal and the swollenness subsided spontaneously. Epipharynx, hypopharynx and larynx were normal at inspection. CRP was 104 mg/L (< 3), ESR 40 mm/hr (< 30) and thyroid function tests were normal. The left part of the thyroid was enlarged, tender and an erythema 10–15 cm in diameter was seen in the anterior neck. Ultrasound revealed a 30x30x30 mm mass in the thyroid with low echogenicity, and a deep puncture for culture and drainage was performed and brownish fluid was extracted. She was treated with oral clindamycin 300 mg tds and oral ciprofloxacin 500 mg bid. The following day she continued to have high fever (38.6 °C). Thyroid surgery was considered. Due to a tick bite a week prior borrelia infection was excluded. Fever, local symptoms and laboratory subsided, she was admitted for four days and antibiotics were continued for in total 10 days. All cultures were negative. A FNA two weeks after discharge showed a colloid cyst, and she had no longer difficulties to swallow. She is still euthyroid without medication four years after this episode.

### Case 4

A 44-year-old woman had a FNA done because of a lump in the right side of the thyroid. Brownish fluid was evacuated, and the lump diminished. Thyroid function tests were normal. Two weeks later the lump increased in size and she presented to Emergency Department. She complained of pain during swallowing, difficulty in eating and experienced dyspnoea when lying down. Fever was fluctuating up to 39 °C. In the right side of the thyroid a 2 × 4 cm hard and tender lump was palpated. CRP was 87 mg/L (< 3), ESR 26 mm/hr (< 20) and leukocytes 12.9 × 10(9)/L (3.5–8.8). A new FNA with deep culture was done from the thyroid and 10 mL brownish fluid was drained. Iv cefuroxime 1.5 g tds was commenced. CT neck showed a 30x27x34 mm mass in the right thyroid lobe. Cultures from blood and thyroid tissue were all negative. After three days, local symptoms had much improved, CRP was 42 mg/L and ESR 29 mm/hr. Antibiotics were changed to po sulfamethoxazole/trimethoprim 800/160 mg bid for another week, and all symptoms subsided. The length of admission was four days. Six month later she represented to Emergency Department with symptoms from the thyroid gland, but the thyroid ultrasound only showed a 14 × 8 mm cyst. She continued to be euthyroid and well four years after the first presentation.

### Case 5

A 68-year-old woman presented to the Emergency Department at a county hospital with dyspnea, atrial flutter, fever (38.4 °C) and a CRP of 426 mg/L (< 4). CT thorax displayed infiltrates in the left lung and signs of heart failure. She developed sepsis and was admitted to the intensive care unit where she was intubated, and later the same day transferred to a tertiary hospital. By that time treatment with iv piperacillin/tazobactam 4 g/0.5 g tds and a single dose of iv garamycin 460 mg had been commenced. Serum calcium was 3.70 mmol/L (2.10–2.55) and parathyroid hormone level (PTH) 594 ng/L (10–73). Antibiotic treatment was continued, and she required noradrenaline to compensate low blood pressure, in addition to amiodarone infusion for atrial flutter. One episode with ventricular fibrillation was cured with cardioversion. Due to continuing respiratory distress tracheotomy was performed after 11 days. From the left thyroid lobe and parotic gland large amounts of pus was drained, with less extent of pus from the lower part of the left lobe. The left thyroid lobe was highly fibrotic in a para pharyngeal abscess. Isthmectomy was performed, but anatomic structures were not identifiable. Cultures from nasopharynx showed airway pathogens, from pus *Candida*, from blood *Streptococci sanguineous*, from pleura *Coagulase negative staphylococci* and later from faeces *Clostridium difficile*. She also had a bilateral pleura empyema which was drained repeatedly. Piperacillin/tazobactam was changed after a week to iv imipenem/cilastatin for three weeks together with iv metronidazole, and later iv vancomycin. The thyroid abscess was drained once again after a week. She was treated at the intensive care unit for 37 days in total and thereafter transferred to a medical ward for another six weeks. PTH continued to rise to 1368 ng/L, whereas serum calcium was maintained normal with cinacalcet 60 mg od. Parathyroid scintigraphy was positive on the left side, which was operated five months later, after she had had an extensive treatment at a Rehabilitation Center. The removed parathyroid gland weighed 0.7 g and was judged by the pathologist as a parathyroid adenoma. The calcium levels normalized, but PTH elevation persisted the following years between 175 to 565 ng/L. CT thorax and neck after parathyroidectomy did not display any remaining abscess, pleural thickening, enlarged lymph glands or parathyroid adenoma. She passed away three years later in asystole, secondary to severe heart failure and pneumonia.

### Case 6

A previously healthy 28-year-old woman had three months after delivery developed left-sided swollenness in the neck, pain, fever and tachycardia in the last 10 days. At another hospital this was suspected as a subacute thyroiditis as TSH was 0.1 mIU/L (0.4–3.5) and free T4 21 pmol/L (10–22). Thyroglobulin was normal. Treatment was commenced with oral prednisolone 25 mg daily and she was discharged after two days. One day later she returned with progression of the lump in the neck and breathing difficulties. She was admitted to the intensive care unit at the tertiary hospital with the combined diagnosis of epiglottitis and acute thyroiditis. At this time there was a non-tender 50 × 50 mm large lump to the side of the throat with no erythema. Heart frequency was 132 beats per minute and temperature 39.3 °C. Increasing edema of the glottis was noted and she could not swallow but had no stridor. Iv cefuroxime 1.5 g tds was initiated. Repeated fiberscope examinations showed progressive edema and she was subsequently intubated. A CT showed a 70 mm large multicystic process in the left thyroid lobe expanding cranially and dorsally dislocating the larynx to the right, as a thyroid abscess, which was drained with three drains. There was an anaerobe smell from the extracted pus, and iv 1000 mg metronidazole od added for four days. Clinically she improved and could leave the intensive care unit after five days, and the hospital after additional three days. Cultures from nasopharynx revealed *Hemophilic influenza*, and from blood *Pepto streptococci*. In the abscess there was also *Hemophilic influenza*. Iv antibiotic treatment was altered to oral metronidazole 400 mg tds for five days and amoxicillin/clavulanic acid 875/125 mg bid for ten days. Thyroid function tests were normalized after one months. There has been no recurrence at the review eight years later.

## Discussion

AST is a very rare infectious disease affecting the thyroid. We describe a relatively large series of six cases in otherwise healthy adult patients without anatomic anomalies with AST. In two iatrogenic causes could be established, in four no clear pathologic route could be established, and no causative organisms could be cultured in two. All of them were treated with antibiotics, intravenous treatment initially in five and oral in one, three were treated with drainage, and all of them recovered the acute episode.

AST is most often described in children with anatomic aberrations or in patients with immunosuppression such as transplant recipients or after chemotherapy. However, the clinical picture may differ between children and adults. With prompt diagnosis and treatment, and in patients without underlying disease, the outcome is very good, although mortality is still reported [29]. In AST, the clinical spectrum is broad, as even asymptomatic cases have been reported [30]. Typically, symptoms in AST are similar to those in patients with subacute or chronic thyroiditis. Other differential diagnosis are hemorrhage in the thyroid, amiodarone induced thyrotoxicosis, infarction of a thyroid nodule [31], and rapidly growing thyroid cancers. Prompt treatment is necessary as the infection may cause destruction of the thyroid and the parathyroid glands, spread to other organs, or cause abscess rupture, vocal cord palsy and fistulae to the trachea or oesophagus [32, 33]. None of these were seen in our six patients.

Among patients > 20 years old in the study by Yu et al. 32/66 were immunocompromised. Their mortality rate was 3.7%, and all these had HIV, cancer or other underlying disease [26]. In contrast, none of our patients were immunocompromised and the only death was not related to AST.

One major route to AST is a pyriform sinus fistula, and if the left lobe is involved the likelihood of an underlying pyriform fistula is increased [34]. Indeed, Miyauchi described this mechanism in 135 of 139 cases in a review [35]. If these patients mainly were children was not clear, although a pyriform fistula can be detected also in adults [7]. In the review by Yu et al. investigating 191 patients with AST 70% had some anatomical structural defect [26]. In contrast, in one study describing 10 adult patients with AST, only one had a pyriform fistula [36]. Also, our six cases had an even distribution of their AST in both thyroid lobes and no such anatomic defect was found.

Ultrasound, or preferably CT, should be deployed to verify a pyriform fistula but could in an early phase be erroneous. CT have the advantage to give information on extra thyroidal involvement. A barium swallow investigation or endoscopic hypopharyngoscopy performed after the acute phase can prove such a lesion [37, 38]. Branchial arch anomalies are other causes, which is not only seen in children [39]. FNA and cultures confirms the diagnosis, the causative organism(s) and which antibiotic to choose, although responsible agents are not always found, as demonstrated by our Case nr 3 and 4.

The age of patients with AST is not always reported. Our patients had a median age of 51 years at presentation, and in this age group treatment and prognosis could be more favourable when no concurrent disease or anatomical factors are present. If AST has a more dismal effect in less developed countries is not known, as reports from these countries are sparse. There may also be underreporting, and a restraint from publishing negative clinical outcomes, as discussed by Paes et al. [1].

If antibiotic treatment fails, surgery as incision with drainage can be necessary. Drainage has in multiple reports been successful, and can be repeated if the abscess persists, or if there is a deterioration [1, 40]. Drainage is urgent in unstable patients with comprised airways. Open surgery, with total, near total or hemithyroidectomy can in severe cases be deployed to relieve pressure symptoms, and later in patients that do not respond to adequate antibiotic treatment and drainage [11, 41, 42]. Complications to surgery are damage to tissues in the area as the parathyroid glands and the recurrence nerve. As identification of anatomic structures in excessive infection often is difficult [12], these tissues may be impossible to find. Therefore, open surgery should be performed with caution and in selected patients. If there is an anatomic defect, surgery could wait until the abscess has been treated with antibiotic therapy, and often also drainage.

The concomitant hypercalcemia and AST in Case 5 is intriguing. No previous calcium levels were known to us. Cultures implied an infection from the upper airways spreading to the thyroid, and the parathyroid lower left gland. If the patient also had primary hyperparathyroidism and if the infection could activate synthesis of PTH remains unclear, but the latter is purely speculative. The ongoing elevation of PTH post-operatively, without any parathyroid enlargement, could to at least to some extent be a result of vitamin D deficiency. However, no such test was performed.

This study has some limitations like all retrospective studies. The systematic review of all AST cases in our centre may have been incomplete due to not being identified by us because of inadequate ICD-coding, Moreover, no diagnostic investigations such as a barium investigation or hypopharyngoscopy was performed, instead imaging was performed with ultrasound, CT and MRI. Thus, presence of anatomic aberrations cannot entirely be excluded, but as none of our patients had any recurrences during seven years of follow-up anatomic aberration are unlikely.

## Conclusions

Acute suppurative thyroiditis is an extremely rare disease in the adult thyroid. The mainstay of therapy is antibiotic therapy, drainage and in selected cases surgery. With this approach the prognosis is favourable in patients who are immunocompetent, and who does not have branchial arch anomalies or a pyriform fistula.

## Data Availability

All data generated or analyzed during this study are included in this published article.

## Abbreviations

AST: Acute suppurative thyroiditis
CRP: C-reactive protein
CT: Computed Tomography
ESR: Erythrocyte Sedimentation Rate
FNA: Fine needle aspiration biopsy
HIV: Human Immunodeficiency Virus
ICD: 10 International Classification of Diseases version 10
MRI: Magnetic Resonance Imaging
PTH: Parathyroid Hormone
TPOab: Thyroid Peroxidase antibodies
TRAb: Thyroid receptor antibodies
TSH: Thyroid Stimulating Hormone
